# Structure-based Markov random field model for representing evolutionary constraints on functional sites

**DOI:** 10.1186/s12859-016-0948-2

**Published:** 2016-02-24

**Authors:** Chan-Seok Jeong, Dongsup Kim

**Affiliations:** Department of Bio and Brain Engineering, Korea Advanced Institute of Science and Technology (KAIST), 291 Daehak-ro, Yuseong-gu, Daejeon, 34141 Republic of Korea

**Keywords:** Markov random field, Coevolution analysis, Protein structure, Functional site, Catalytic site, Allosteric site

## Abstract

**Background:**

Elucidating the cooperative mechanism of interconnected residues is an important component toward understanding the biological function of a protein. Coevolution analysis has been developed to model the coevolutionary information reflecting structural and functional constraints. Recently, several methods have been developed based on a probabilistic graphical model called the Markov random field (MRF), which have led to significant improvements for coevolution analysis; however, thus far, the performance of these models has mainly been assessed by focusing on the aspect of protein structure.

**Results:**

In this study, we built an MRF model whose graphical topology is determined by the residue proximity in the protein structure, and derived a novel positional coevolution estimate utilizing the node weight of the MRF model. This structure-based MRF method was evaluated for three data sets, each of which annotates catalytic site, allosteric site, and comprehensively determined functional site information. We demonstrate that the structure-based MRF architecture can encode the evolutionary information associated with biological function. Furthermore, we show that the node weight can more accurately represent positional coevolution information compared to the edge weight. Lastly, we demonstrate that the structure-based MRF model can be reliably built with only a few aligned sequences in linear time.

**Conclusions:**

The results show that adoption of a structure-based architecture could be an acceptable approximation for coevolution modeling with efficient computation complexity.

## Background

Coevolution analysis is widely used to model the interdependency between protein residues in a multiple sequence alignment (MSA). Since it is generally believed that highly correlated mutation patterns represent evolutionary constraints resulting from structural or functional aspects [1], coevolutionary information has been widely used to describe residue-residue contacts [2], sequence comparisons [3], deleterious substitutions [4], drug-resistant positions [5], various types of functional sites [6, 7], allosteric signaling pathways [8], protein-protein interactions [9], and for protein design [10].

Despite the usefulness of coevolution information, its accurate estimation remains challenging because of various noise factors such as those derived from phylogenetic signals [11], indels [12] and indirect signals [13]. Recently, new coevolution analysis methods have been developed that are based on a type of probabilistic graphical model called the Markov random field (MRF), which have shown remarkable improvements for estimation [9, 14–19]. Unlike the earlier approaches based on local estimates [12, 20–22], the MRF methods utilize a global sequence context of multiple alignment, and thus can effectively overcome interference from indirect signal noise.

All of the MRF methods are broadly similar to each other with respect to graphical modeling and coevolution estimations. They represent an MSA as a graphical model—in which each node encodes a distribution of amino acids at a specific residue position, and each edge encodes a joint distribution of amino acids between two connected residues—and coevolution scores are calculated from the edge weights. However, because parameterization using a likelihood function is computationally challenging, recent studies have suggested different methods for learning a MRF model. GMRC [14] uses a greedy structure search that develops a graphical architecture by iteratively updating the edge set. mpDCA [9] and mfDCA [15] approximate the likelihood function by using a message-passing algorithm and a mean-field equation, respectively. PSICOV [17] uses a sparse inverse covariance estimation technique with a graphical LASSO penalty instead of directly computing the MRF model. Most recently, some methods have been proposed to replace the likelihood function with an alternative objective function, which is more tractable [16, 19]. In particular, GREMLIN has shown the most advanced performance, which relies on a pseudo-likelihood objective and parameter regularization [18]. Nevertheless, the use of MRF methods has not been comprehensively assessed with respect to the functional aspect of the coevolutionary constraint; instead, most of these assessments have thus far focused on the ability for protein structure prediction. Moreover, the accuracy of MRF methods considerably depends on the number of sequences comprising the MSA [23, 24].

In this paper, we present a structure-based MRF (SMRF) model whose graphical architecture is determined by using the protein structure information, and then derive a novel positional coevolution estimate using the node weight. We further apply the SMRF model to three data sets with different types of functional annotations, and demonstrate the association between coevolution information and functional sites. In addition, we examine the computational robustness and efficiency of the proposed SMRF-based coevolution analysis.

## Methods

### Structure-based Markov random field

#### Overview of the Markov random field model

To estimate the evolutionary constraints on functional sites, we use the MRF approach as a class of probabilistic graphical models represented as an undirected graph. Similar to a Bayesian network, the nodes of an MRF represent the variables, and the edges represent direct dependency between the variables of the neighboring nodes. However, an MRF is defined on the basis of undirected graphs and may be cyclic. This distinctive feature enables the MRF to model certain dependencies such as the symmetric influence of neighboring variables, whereas a Bayesian network forces a directionality for the interactions. Recent studies [9, 14–19] have shown that MRF methods are suitable for modeling coevolutionary relationship between residues of a protein in an MSA.

#### Modeling of an multiple sequence alignment

An MRF model describing evolutionary information can be built from the MSA by representing the residues of a target protein with nodes. Therefore, the individual distribution of amino acids at a specific residue *i* is encoded to the node weight *ϕ*_*i*_ defined as

$$\begin{array}{*{20}l} \phi_{i} =\left[e^{v_{i}(x_{1})} e^{v_{i}(x_{2})} \dotsm e^{v_{i}(x_{K})}\right] \text{,} \end{array} $$

where $e^{v_{i}(x_{k})}\phantom {\dot {i}\!}$ represents the distribution of amino acid *k* at column *i* of the MSA. Likewise, the probabilistic interaction between residues *i* and *j* is encoded to the corresponding edge weight *ψ*_*ij*_ defined as

$$\psi_{ij} = \left[ \begin{array}{cccc} e^{w_{ij}(x_{1}, x_{1})}& e^{w_{ij}(x_{1}, x_{2})}& \dotsm& e^{w_{ij}(x_{1}, x_{K})} \\ e^{w_{ij}(x_{2}, x_{1})}& e^{w_{ij}(x_{2}, x_{2})}& \dotsm& e^{w_{ij}(x_{2}, x_{K})} \\ \vdots& \vdots& \ddots& \vdots \\ e^{w_{ij}(x_{K}, x_{1})}& e^{w_{ij}(x_{K}, x_{2})}& \dotsm& e^{w_{ij}(x_{K}, x_{K})} \end{array} \right] \text{,} $$

where $\phantom {\dot {i}\!}e^{w_{ij}(x_{k_{i}}, x_{k_{j}})}$ represents the joint distribution of amino acid *k*_*i*_ at column *i* and amino acid *k*_*j*_ at column *j* of the MSA. Because we consider a gap as an additional amino acid, *k* is set to 21, and then the node and edge weights consist of a 21 and 21-by-21 dimensional vector and matrix, respectively.

Given a set of parameters for the node and edge weights, the likelihood of an aligned sequence *x* can be written as

$$P(x) = \frac{1}{Z} \prod_{i} \phi_{i}(x_{i}) \prod_{i, j} \psi_{ij}(x_{i}, x_{j}) \text{,} $$

where *x*_*i*_ represents the matched amino acid at column *i* of the MSA, and *ϕ*_*i*_(*x*_*i*_) and *ψ*_*ij*_(*x*_*i*_,*x*_*j*_) denote $\phantom {\dot {i}\!}e^{v_{i}(x_{i})}$ and $\phantom {\dot {i}\!}e^{w_{ij}(x_{i}, x_{j})}$, respectively. *Z* denotes the partition function to convert the following terms into probability. Thus, the summation of *P*(*x*) over all possible *x* values becomes 1.

#### Determining the Markov random field architecture from intramolecular contact

**Fig. 1:**
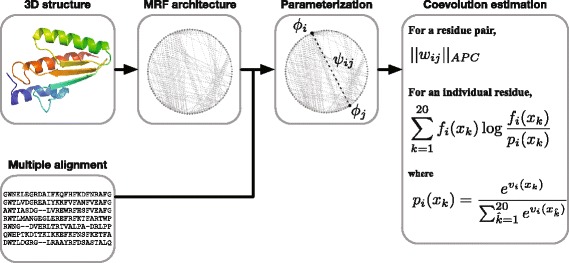
Overview of the procedure for building a structure-based Markov random field (MRF) model and quantifying coevolutionary constraints. The MRF architecture is determined from the three-dimensional structure of a target protein. Each residue of the target protein is represented as a node, and each intramolecular contact is represented as an edge connecting the corresponding residue nodes. After the graphical topology is determined, parameterization is performed for the multiple sequence alignment in the same way as in conventional MRF methods. Then, pairwise and positional coevolution scores are calculated from the edge and node weights, respectively

To effectively describe the coevolutionary constraint for biological functionality, we determine the MRF architecture based on the three-dimensional protein structure. As shown in Fig. 1, the edges of the SMRF model are defined for the residue pairs in contact. Thus, the topology of the graph for the SMRF model becomes sparse, in contrast to the complete graph topology of a conventional MRF model such as GREMLIN. Assuming that residues in contact are more likely to interact with each other due to their spatial proximity, the structure-based architecture would be a computationally efficient and biochemically relevant representation. More importantly, because the edges of the SMRF have similar constraints in terms of the protein structure, the coevolution signals encoded on the edges would be differentiated according to other types of constraints such as biological functionality. In the present study, intramolecular contact is determined by the C_*β*_- C_*β*_ (C_*α*_ in the case of GLY) atomic distance of <8 Å, by following the contact definition adopted in the CASP experiment [25].

#### Parameterization procedure

Given the MSA $\mathcal {D} = \lbrace x[\!1], x[\!2], \dotsm, x[\!M] \rbrace $ consisting of *M* homologous sequences, the parameters for the MRF can be estimated by maximizing the log-likelihood sum written as

$$\text{ll}(v, w | \mathcal{D}) = \sum_{m} \log P(x[\!m]) \text{.} $$

However, because the partition function *Z* is defined over the entire space of the node and edge weights, obtaining the parameters by the maximum log-likelihood is computationally intractable. Alternatively, similar to GREMLIN, we use the pseudo-likelihood objective, which replaces the likelihood *P*(*x*) with $\prod _{i} P(x_{i} | x_{-i})$, where *x*_−*i*_ represents $x_{1}, \dotsm, x_{i-1}, x_{i+1}, \dotsm, x_{K}$, and *P*(*x*_*i*_|*x*_−*i*_) is defined as

$$P(x_{i} | x_{-i}) = \frac{1}{Z_{i}} \phi_{i}(x_{i}) \prod_{j} \psi_{ij}(x_{i}, x_{j}) \text{,} $$

where *Z*_*i*_ represents the local partition function that is computationally more tractable. Consequently, the pseudo-likelihood objective function is defined as

$${\fontsize{7.8}{8}{\begin{aligned} &\text{pll}(v, w | \mathcal{D}) \\ &\quad= \sum_{m} \sum_{i} \log P(x_{i}[\!m] | x_{-i}[\!m]) \\ &\quad= \sum_{m} \sum_{i} \left[ \log \phi_{i}(x_{i}[\!m]) + \sum_{j} \log \psi_{ij}(x_{i}[\!m], x_{j}[\!m]) - \log Z_{i} \right], \\ &\quad= \sum_{m} \sum_{i} \left[ v_{i}(x_{i}[\!m]) + \sum_{j} w_{ij}(x_{i}[\!m], x_{j}[\!m]) - \log Z_{i} \right] \text{.} \end{aligned}}} $$

In addition to the pseudo-likelihood objective, to avoid over-fitting, we use *L*_2_-regularization, defined as

$$R(v, w) = \lambda_{v} \sum_{i} \| v_{i} {\|_{2}^{2}} + \lambda_{w} \sum_{i, j} \| w_{ij} {\|_{2}^{2}} \text{,} $$

where *λ*_*v*_ and *λ*_*w*_ are determined as 0.01 and 0.2, respectively, as in the GREMLIN model.

To minimize the objective function, $R(v, w) - \text {pll}(v, w | \mathcal {D})$, we use the limited memory Broyden-Fletcher-Goldfarb-Shannon (L-BFGS) algorithm [26] of libLBFGS implementation [27]. Compared to the GREMLIN model, an SMRF model can be built more efficiently, despite the similarity of their parameterization procedures, because the structure-based graph topology of the SMRF effectively reduces the search space for edge weights.

### Measurement of evolutionary constraints

The coevolution score between residues *i* and *j* is calculated by using the norm of the edge weight, defined as

$$\sum_{k_{i}=1}^{20} \sum_{k_{j}=1}^{20} \left[w_{ij}(x_{k_{i}}, x_{k_{j}})\right]^{2} \text{,} $$

and is corrected by using average-product correction (APC) [28]. Because functionality annotation is usually determined for individual residues, the coevolution score defined for a residue pair should be transformed to a positional value. The proximity-based average is a conventional approach to derive a positional coevolution score. This calculates the average of the coevolution scores between a target residue and its neighboring residues in the three-dimensional structure. Calculating the fraction of coevolution is another approach to determine the positional coevolution value. This involves calculating the fraction of coevolving residue pairs for a target position. According to a previous study [7], we here consider a residue pair to be coevolved if its Z-score of the coevolution score exceeds 3.

Here, we develop a novel estimate of the positional coevolution score by using the node weight of the SMRF model. Since an MRF model tends to factorize the distribution of a random variable at a specific node according to the dependencies with neighboring nodes, the difference between the node weight and the amino acid frequency at a specific position could represent the degree of the coevolutionary constraint for the residue. Based on this assumption, we formulate the positional coevolution score at position *i* by estimating the KL-divergence, defined as

$$\text{NW} = \sum_{k=1}^{20} f_{i}(x_{k}) \log \frac{f_{i}(x_{k})}{p_{i}(x_{k})} \text{,} $$

where *f*_*i*_(*x*_*k*_) represents the amino acid frequency at column *i* of the MSA, and *p*_*i*_(*x*_*k*_) represents the amino acid frequency, defined as

$$p_{i}(x_{k}) = \frac{e^{v_{i}(x_{k})}}{\sum_{\hat{k}=1}^{20} e^{v_{i}(x_{\hat{k}})}} \text{.} $$

Additionally, the conventional conservation score of residue *i* is calculated by using the KL-divergence as

$$\text{KLD}_{f,q} = \sum_{k=1}^{20} f_{i}(x_{k}) \log \frac{f_{i}(x_{k})}{q(x_{k})} \text{,} $$

where *q*(*x*_*k*_) represents the overall amino acid frequency in the MSA. Similar to the KL-divergence, the JS-divergence is used for quantifying the degree of conservation as

$$\text{JSD}_{f,q} = \lambda \text{KLD}_{f,r} + (1-\lambda) \text{KLD}_{q,r} \text{,} $$

where *r* and *λ* are defined as *r*=*λf*+(1−*λ*)*q* and *λ*=0.5, respectively, according to a previous study [29].

To normalize the different distributions, the coevolution and conservation scores are transformed to Z-scores over all of the residues of the protein, respectively.

### Data sets

We built three data sets, each of which describes different types of functional site information. The first data set was collected from the Catalytic Site Atlas (CSA) database [30], which annotates catalytic sites from the literature according to homology. For the CSA data set, the proteins with five or more catalytic sites annotated in the literature were collected. The second data set was collected from the AlloSteric Database (ASD) [31], which annotates allosteric sites from the literature. For the ASD data set, the proteins with five or more annotated allosteric sites were collected. The third data set was collected from the InterPro database [32], which provides comprehensive information on various types of functional sites. For the InterPro data set, the proteins with five or more annotated functional sites were collected. Next, we chose proteins whose Protein Data Bank structure has been determined by X-ray diffraction with a resolution of ≤2.5 Å. To remove sequence redundancy in the data set, the amino acid sequences of proteins were clustered to the maximum sequence identity of <50 % by running the CD-HIT [33]. In addition, for reliably parameterizing the SMRF models, we chose only proteins whose MSA consists of more than 300 sequences. The MSAs were constructed by running the HHblits [34] with the option “-e 0.001” for the NR20 sequence database (last update August 2011) downloaded from the HHblits webpage. The NR20 is the NCBI non-redundant database clustered to the maximum sequence identity of 20 %. Finally, the CSA data set consisted of 99 proteins with 628 catalytic sites, the ASD data set consisted of 54 proteins with 501 allosteric sites, and the InterPro data set consisted of 688 proteins with 15,607 functional sites.

### Assessment

The central objective of our study was to use the structure information for MRF-based coevolution modeling, and to derive a novel positional coevolution estimate that could more accurately represent functional constraints. To assess the effectiveness of the protein structure-based MRF architecture, we compared the SMRF approach with the state-of-the-art MRF methods GREMLIN [18] and PSICOV [17] using the recommended default options. Additionally, we built a random predictor by randomly permutating the coevolution scores of the SMRF 100 times and calculating the average. Since GREMLIN and PSICOV were originally developed to predict residue-residue contacts similar to other previously published MRF methods [9, 14–16, 19], they can only provide scores determined for a residue pair. However, the pairwise coevolution score is not commensurable with the functional annotation determined for an individual residue. Therefore, we computed the GREMLIN-style scores derived from the edge weights of the SMRF model, and compared them with the original GREMLIN and PSICOV scores. With this approach, a positive example is defined as a residue-residue contact composed of at least one functional site, a negative example is defined as a residue-residue contact with no functional site, and residue pairs not in contact are ignored.

The CSA and ASD data sets contain functional sites only covering catalytic and allosteric functionalities, respectively, whereas the InterPro data set provides comprehensive functionality information, which enables a more systematic evaluation. Therefore, for the InterPro data set, we evaluated the association between the positional coevolution score and functional sites by using receiver operating characteristic (ROC) curves that visually describe the relative trade-offs between the true positive rate (TPR) and false positive rate (FPR), where TPR and FPR are defined as

$$\begin{array}{*{20}l} TPR &= \frac{TP}{TP+FN} \\ FPR &= \frac{FP}{FP+TN} \text{,} \end{array} $$

where TP, FP, TN, and FN represent the number of true positive, false positive, true negative, and false negative predictions at a certain cutoff. We compared the overall performance by vertically averaging the ROC curves of the target protein. Moreover, to evaluate the performance for each target protein, we used the area under the ROC curve (AUC), where an AUC value of 1 indicates perfect prediction, 0.5 indicates a random prediction, and <0.5 indicates worse than random. ROC curves and AUC scores were estimated using the ROCR package [35].

## Results

### Evaluation of the protein structure-based architecture

For the SMRF model, we first determined the MRF architecture by describing individual residues and their intramolecular contacts as nodes and edges, respectively, and then calculated the coevolution scores by parameterizing the MRF model. We assumed that a coevolution model explicitly encoding the protein structure could provide a better representation of functional constraints rather than only encompassing structural constraints. To validate this assumption, we examined the association between the coevolution scores and functional sites, and compared the results to those of the conventional MRF method without a structure-based architecture. We used GREMLIN [18], which incorporates the MRF architecture of a complete graph topology connecting all available residues. Except for the MRF architecture, GREMLIN and SMRF calculated the coevolution scores in the same way. For GREMLIN, the coevolution scores for intramolecular contacts were considered. Consequently, the SMRF and GREMLIN scores differed only with respect to the network architecture of the MRF models. In addition, we used PSICOV [17], which utilizes sparse inverse covariance estimation. Similar to GREMLIN, only the coevolution scores of PSICOV for intramolecular contacts were considered.

**Fig. 2:**
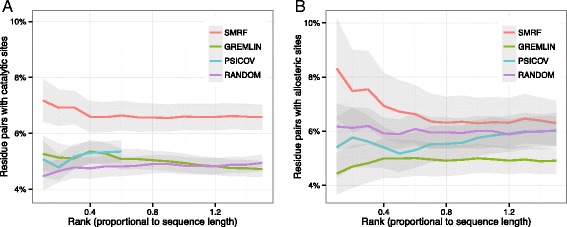
Association of pairwise coevolution scores and functional sites. **a** For the CSA data set, the fraction of residue pairs with at least one catalytic site among hits is represented across the rank cutoff. **b** For the ASD data set, the fraction of residue pairs with at least one allosteric site among hits is represented across the rank cutoff. In (**a**) and (**b**), the rank is normalized by the sequence length of the target protein. For example, the normalized ranks 0.1, 0.2, and 0.5 are equivalent to the top-L/10, top-L/5, and top-L/2, where L is the sequence length of the target protein. The gray shadow represents the standard deviation

We estimated the fraction of residue pairs involving functional sites (RPF) among hits. First, for the CSA data set, the functional sites were determined as catalytic sites. As shown in Fig. 2a, 6.5–7.2 % of the SMRF hits were associated with catalytic sites in the normalized rank of 0.1–1.5, whereas GREMLIN showed an RPF rate of 4.7–5.3 % in the same range. For PSICOV, an RPF rate of 4.8–5.3 % was obtained in the normalized rank of 0.1–0.6. The results for PSICOV are omitted above the normalized rank of >0.6, because the output did not show hits for some of the target proteins in this higher range. The random prediction yielded an RPF rate of 4.5–4.9 %. Therefore, compared to the random prediction, SMRF could increase the RPF rate by up to 33.0–60.8 %, while GREMLIN and PSICOV could only increase the RPF rate by up to 18.1 % and 13.7 %, respectively. Consequently, by using the proximity-based MRF architecture, the coevolution scores were more likely to be associated with catalytic sites.

Next, for the ASD data set, the functional sites were determined as allosteric sites. As shown in Fig. 2b, the SMRF resulted in a higher RPF rate than GREMLIN and PSICOV in the normalized rank of 0.1–1.5, with a rate of 6.3–8.3 %. On the other hand, GREMLIN and PSICOV showed RPF rates of 4.4–5.0 % and 5.2–6.0 %, respectively, which are lower than the RPF rate obtained from the random prediction (5.9–6.2 %). This implies that the coevolution scores of GREMLIN and PSICOV are not associated with allosteric sites, whereas those of SMRF are more likely to correspond to the allosteric sites as well as catalytic sites.

### Evaluation of the positional coevolution measure

In contrast to the coevolution score determined for a residue pair, functionality is generally determined for an individual residue. The conventional methods used to convert the coevolution score for a residue pair to the positional coevolution score are averaging coevolution scores across neighboring links, denoted as EW, or calculating the fraction of strongly coevolving residue pairs, denoted as FC [7, 36–38]. Here, we propose a novel measure of calculating the positional coevolution score by utilizing the node weight of the MRF model, denoted as NW, and investigate the association with functional sites.

**Fig. 3:**
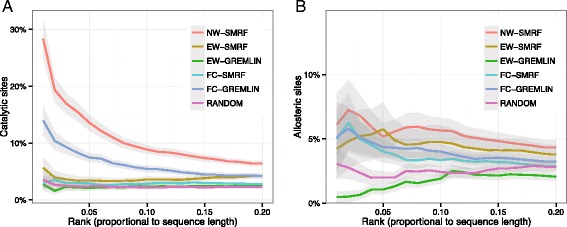
Association of positional coevolution scores and functional sites. **a** For the CSA data set, the fraction of catalytic sites among hits is represented across the rank cutoff. **b** For the ASD data set, the fraction of allosteric sites among hits is represented across the rank cutoff. In (**a**) and (**b**), the rank is normalized by the sequence length of the target protein. For example, the normalized ranks 0.1, 0.2, and 0.5 are equivalent to the top-L/10, top-L/5, and top-L/2, where L is the sequence length of the target protein. The gray shadow represents the standard deviation

First, for the CSA data set, we investigated the association between positional coevolution scores and catalytic sites, as shown in Fig. 3a. When comparing EW measures, SMRF showed a higher fraction of catalytic sites than GREMLIN, analogous to the results described above. On the other hand, when comparing FC measures, GREMLIN showed a higher fraction of catalytic sites than SMRF. However, when using the NW measure, SMRF identified many more catalytic sites. In particular, at the normalized rank of 0.01, NW-SMRF showed an average of 28.4 % catalytic sites, while EW-SMRF, EW-GREMLIN, FC-SMRF, and FC-GREMLIN showed fractions of 5.6 %, 2.7 %, 3.0 %, and 14.0 %, respectively. In addition, the fraction of catalytic sites from NW-SMRF was 7.9-times higher than that of the random prediction. Below the normalized rank of <0.05, NW-SMRF still showed a fraction of catalytic sites that was >5.8-times higher than that of the random prediction.

Next, for the ASD data set, we investigated the association between positional coevolution scores and allosteric sites, as shown in Fig. 3b. Similar to the above result, EW-SMRF and FC-GREMLIN showed higher fractions of allosteric sites than EW-GREMLIN and FC-SMRF, respectively. Furthermore, for the most part, NW-SMRF showed the highest fraction of allosteric sites below the normalized rank of <0.2. In the normalized rank of 0.01–0.05, NW-SMRF showed a fraction of allosteric sites of 5.2–6.1 %, which is 1.5–3.0-times higher than that of the random prediction.

**Fig. 4:**
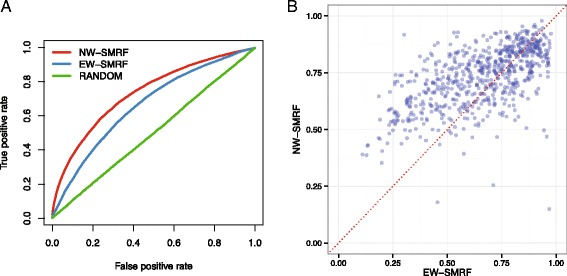
Performance comparison of positional coevolution measures of SMRF methods. **a** The solid lines represent the ROC curves vertically averaged over all of the target proteins. NW-SMRF and EW-SMRF represent the positional coevolution measures derived from the node and edge weights, respectively. **b** Scatter plot of AUC values for NW-SMRF (y-axis) and EW-SMRF (x-axis)

In addition to the CSA and ASD data sets, we evaluated the performance of the NW measure by estimating the ROC curve for the InterPro data set comprised of a comprehensive annotation of residue functionality. When comparing the ROC curves, the NW-SMRF was found to outperform EW-SMRF, as shown in Fig. 4a. The AUC values were 0.733 and 0.663 for NW-SMRF and EW-SMRF, respectively, indicating that the AUC values improved by 10.5 %. In addition, we compared the AUC values of NW-SMRF and EW-SMRF for each target protein. As shown in Fig. 4b, NW-SMRF showed significant improvement, with a *p*-value of <2.2e-16 (Wilcoxon signed-rank test), outperforming EW-SMRF for 64.0 % of the targets. Consequently, from the results for the CSA, ASD, and InterPro data sets, the novel positional coevolution score based on the node weight of MRF can effectively estimate the evolutionary constraints associated with various types of functional sites.

### Effectiveness of positional coevolution information in combination with conservation information

**Fig. 5:**
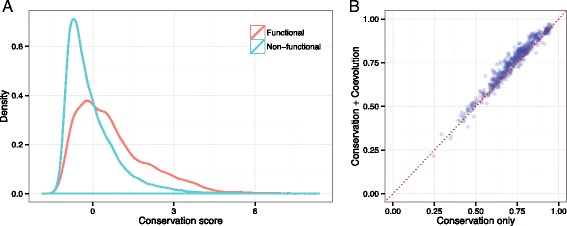
Combination of positional coevolution and conservation information. **a** Kernel density estimate of the conservation score distribution represented for functional and non-functional sites. **b** Scatter plot of AUC values for the logistic regression model combining conservation and coevolution scores (y-axis) and the conservation score-based prediction (x-axis)

Since conservation information represents the primary evolutionary constraint and coevolution information represents higher-order evolutionary constraints, combining this information could be useful to gain a better estimation, owing to mutual compensation between the types of information. To verify the complementary characteristics of coevolution and conservation information, we investigated the density distribution of conservation scores for functional and non-functional sites. As shown in Fig. 5a, the densities of conservation scores for functional and non-functional sites were reversed for conservation score values close to 0. This indicates that prediction of a residue to a functional site is more likely to fail when the conservation score is <0. Hence, using only the conservation score could easily lead to neglecting functional sites with conservation scores of <0, which comprised 35.9 % of all functional sites in the InterPro data set. Moreover, because functional site prediction is commonly performed for a whole set of residues of a given target protein, and a small fraction of residues belong to functional sites, the sensitivity of the conservation score may decrease considerably. Thus, additional information to supplement conservation could be useful for increasing prediction of constraints.

To validate the effectiveness of combining the positional coevolution measure with conservation information, we calculated conservation scores for protein residues by using the KL-divergence from the MSA, and built logistic regression models that use both conservation and coevolution scores as input features. For the InterPro data set, we conducted 20-fold cross-validation 30 times, and averaged the AUC values for each target. The 20-fold cross-validation procedure was carried out by randomly splitting targets into 20 subsets, and predicting functional sites for each subset with a predictive model that learned from the remaining 19 subsets. The AUC values obtained by combining coevolution and conservation scores were compared against the AUC values obtained when using only the conservation scores. As shown in Fig. 5b, combining the coevolution and conservation scores improved the predictive power for 85.3 % of the target proteins with a *p*-value of <2.2e-16 (Wilcoxon signed-rank test). In addition, we replaced the KL-divergence with the JS-divergence, because a previous study showed that JS-divergence outperformed KL-divergence for functional site predictions [29]. The overall AUC statistics are summarized in Table 1, suggesting that positional coevolution information could consistently complement conservation information obtained from different types of measurements. Similar to the combination with KL-divergence, combining coevolution scores with JS-divergence conservation scores improved the predictive power for 84.5 % of the target proteins (Wilcoxon signed-rank test *p*-value <2.2e-16).

**Table 1:** Average AUC values of the logistic regression model for the InterPro data set

Feature	AUC _0.1_	AUC _0.2_	AUC _0.5_	AUC
NW-SMRF	0.023	0.069	0.275	0.733
KLD	0.024	0.071	0.277	0.733
KLD + NW-SMRF	**0.027**	**0.076**	**0.293**	**0.758**
JSD	0.025	0.072	0.279	0.736
JSD + NW-SMRF	**0.027**	**0.077**	**0.294**	**0.758**

AUC _0.1_, AUC _0.2_, and AUC _0.5_ represent the partial area under the ROC curve spanning the false positive rates of 0.1, 0.2, and 0.5, respectively. The best AUC value between the conservation score and the combination of the conservation score with the coevolution score is shown in bold. NW-SMRF represents the coevolution measure derived from node weights. KLD and JSD represent conservation measures using KL-divergence and JS-divergence, respectively. KLD + NW-SMRF and JSD + NW-SMRF represent the logistic regression models combining the coevolution and conservation measures

Furthermore, we validated the effectiveness of positional coevolution information in combination with conservation information for the CSA and ASD data sets to investigate how well the combination could perform with specific types of functional-site data sets. Similar to the results for the InterPro data set, 20-fold cross-validation was carried out 30 times, and the AUC values were averaged for each target. As shown in Tables 2 and 3, the combined methods consistently improved the individual conservation measures for both the CSA and ASD data sets, despite the fact that catalytic and allosteric sites are known to have different conservation characteristics. Overall, these results demonstrate that the novel positional coevolution score consistently complements the conventional conservation score for various types of functional sites.

**Table 2:** Average AUC values of the logistic regression model for the CSA data set

Feature	AUC _0.1_	AUC _0.2_	AUC _0.5_	AUC
NW-SMRF	0.030	0.080	0.288	0.736
KLD	0.047	0.115	0.362	0.839
KLD + NW-SMRF	**0.049**	**0.120**	**0.372**	**0.851**
JSD	0.045	0.110	0.353	0.829
JSD + NW-SMRF	**0.048**	**0.116**	**0.365**	**0.844**

See Table 1 for a description of the statistics

**Table 3:** Average AUC values of the logistic regression model for the ASD data set

Feature	AUC _0.1_	AUC _0.2_	AUC _0.5_	AUC
NW-SMRF	0.012	0.040	0.189	0.607
KLD	0.013	0.041	0.196	0.607
KLD + NW-SMRF	0.013	**0.042**	**0.202**	**0.618**
JSD	0.012	0.039	0.187	0.594
JSD + NW-SMRF	0.012	**0.041**	**0.194**	**0.609**

See Table 1 for a description of the statistics

Next, we compared the coefficients of the logistic regression model to investigate the contribution of each term according to a specific functionality. In this case, the logistic regression models were built using the whole target samples for the InterPro, CSA, and ASD data sets. As shown in Table 4, both the coevolution and conservation terms showed a significant contribution for functional site prediction. However, the amount of the contribution differed for each data set. Compared to the InterPro data set, which consists of comprehensively determined functional sites, the CSA data set, which is specialized for catalytic function, showed a higher correlation with conservation information, whereas the ASD data set, which is specialized for allosteric function, showed a lower correlation with conservation information. Therefore, combining the positional coevolution and conservation scores according to a specific type of functionality would more accurately represent the evolutionary constraints acting at the relevant sites of interest.

**Table 4:** Weight values for coevolution (NW-SMRF) and conservation (KLD) terms, and intercept values of logistic regression models for the InterPro, CSA, and ASD data sets

Data set	NW-SMRF	KLD	Intercept
InterPro	0.241^***^	0.446^***^	−3.033^***^
CSA	0.226^***^	0.875^***^	−4.638^***^
ASD	0.125^*^	0.195^**^	−3.775^***^

*p*-values: ***(<2.2e-16), **(3.5e-5), *(0.003)

### Robustness against the size of the multiple sequence alignment

**Fig. 6:**
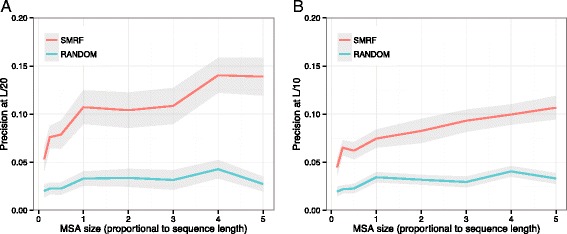
Dependence of prediction on the number of aligned sequences in the multiple sequence alignment (MSA). For 47 target proteins, **a** the precisions, determined as the fractions of catalytic sites, at the rank cutoff of **a** top-L/20 and **b** top-L/10 (y-axis) are represented according to MSAs of different sizes (x-axis). The gray shadow represents the standard deviation

Recent studies [18] have suggested that for a meaningful MRF analysis, the number of aligned sequences in an MSA should exceed five times the length of the target sequence. However, these studies were based on the MRF model with a complete graph topology. In the present study, we reduced the topological complexity by using the three-dimensional protein structure, and the resulting SMRF model had fewer edge parameters. Accordingly, the SMRF model can be built with a smaller number of aligned sequences. To demonstrate the robustness of the SMRF to variations in the MSA size, for the CSA data set, we chose 47 proteins with an MSA size of >5L, where L indicates the sequence length of the target protein, and then generated sub-MSAs by randomly including aligned sequences. Because the EW and FC measures performed poorly in the analyses described above, we examined only the positional coevolution scores, NW-SMRF, which were compared to the random prediction. As shown in Fig. 6, SMRF only showed a moderate difference in performance in the range of >1L, but performance considerably decreased for MSA sizes of <1L. Therefore, for a significant coevolution analysis, SMRF may require less aligned sequences than conventional MRF methods.

### Computational complexity

**Fig. 7:**
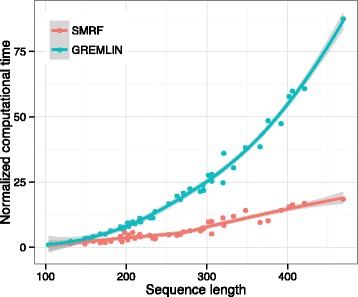
Evaluation of computational complexity. For 47 target proteins, the computation time for running the GREMLIN and SMRF methods is represented according to the sequence length of the target. Each computation time is scaled by the computation time of the shortest target, 1dco_A. Solid lines represent the local fitting lines for the GREMLIN and SMRF methods. The gray shadow represents the standard deviation

The use of a structure-based architecture in SMRF can reduce computation complexity. Conventional MRF methods with a complete graph topology have computation complexity that is proportional to the square of the length of the target sequence. However, because SMRF only considers residue pairs in contact, its computation complexity increases linearly with respect to the number of intramolecular contacts, which is usually proportional to the target sequence length. We compared the computation time spent for building the MRF model. The same 47 proteins chosen for the robustness assessment described above were used with the sub-MSA of a size of 5L. Because GREMLIN and SMRF were implemented in different environments, including different programming languages and optimization levels, we scaled the computation times by that of the shortest sequence, 1dco_A, consisting of 104 amino acids (aa). As shown in Fig. 7, as the target length increased, GREMLIN showed a quadratic increase in computation time, but SMRF showed a linear increase. For instance, for the targets 1tyf_A (193 aa) and 1dli_A (402 aa), the computation times of GREMLIN were 2.66- and 3.71-times higher than those of SMRF, respectively. This result indicates that SMRF can efficiently reduce the computation complexity.

## Discussion and conclusion

The effectiveness of SMRF for modeling evolutionary constraints derives from the fact that the graph topology is determined according to the proximity of protein residues. Explicitly encoding intramolecular contacts forces the MRF edges to share similar structural constraints, so that the edges become parameterized along other sorts of biochemical constraints, including those related to functional significance. Moreover, this approach could avoid the potential bias of a covariation measure to the core region [39], and improve the signal-to-noise ratio of coevolution information. Consequently, in conjunction with MRF methodology that considers the global context of a random variable distribution, SMRF can encode the evolutionary information associated with the functional aspect. Based on comparisons with the conventional MRF method, we have demonstrated that use of an MRF architecture derived from the three-dimensional protein structure can enhance the ability to derive information about the inter-dependencies among functional residues.

Although the edge weight of the MRF model has been commonly used for coevolution analysis, the node weight has not been utilized sufficiently. In the present work, we developed a novel positional coevolution estimate by using the node weight of the SMRF model. This positional coevolution score has a form comparable with a traditional conservation estimate; thus, the integrated analysis of coevolution and conservation information can be easily achieved. Moreover, various machine-learning methods could append the positional coevolution score as an additional component of their feature vector.

The use of a structure-based architecture in this context is particularly advantageous when there are insufficient available sequences for carrying out the conventional MRF method. Previous studies have suggested that an MSA consisting of more than 5L sequences [18] or 1000 sequences [24] is required for reliable coevolution analysis. However, in this paper, we demonstrated that the SMRF method could perform robustly for an MSA with fewer aligned sequences, which could extend the applicability of coevolution analysis. Although high-throughput sequencing progress is continuously expanding sequence databases, information on certain kinds of proteins such as newly evolved or rarely populated proteins has not been expanded from this technology. Therefore, the extended applicability of the method proposed herein could be useful for large-scale coevolution studies.

## Availability of supporting data

The data set supporting the results presented in this article is available in the Zenodo repository (http://dx.doi.org/10.5281/zenodo.32989). The repository holds 1) the structures, sequences, multiple alignments, and functionality annotations for proteins of the CSA, ASD, and InterPro data sets; and 2) the pairwise and positional coevolution scores.

## Software availability

Project name: SMRF Project home page: https://github.com/jeongchans/smrfArchived version: http://dx.doi.org/10.5281/zenodo.45543Programming language: Python License: MIT
